# Rac1 modulates the formation of primordial follicles by facilitating STAT3-directed Jagged1, GDF9 and BMP15 transcription in mice

**DOI:** 10.1038/srep23972

**Published:** 2016-04-06

**Authors:** Lihua Zhao, Xinhua Du, Kun Huang, Tuo Zhang, Zhen Teng, Wanbao Niu, Chao Wang, Guoliang Xia

**Affiliations:** 1 State Key Laboratory of Agrobiotechnology, College of Biological Sciences, China Agricultural University, Beijing, 100193, China

## Abstract

The size of the primordial follicle pool determines the reproductive potential of mammalian females, and establishment of the pool is highly dependent on specific genes expression. However, the molecular mechanisms by which the essential genes are regulated coordinately to ensure primordial follicle assembly remain a mystery. Here, we show that the small GTPase Rac1 plays an indispensable role in controlling the formation of primordial follicles in mouse ovary. Employing fetal mouse ovary organ culture system, we demonstrate that disruption of Rac1 retarded the breakdown of germline cell cysts while Rac1 overexpression accelerated the formation of primordial follicles. In addition, *in vivo* inhibitor injection resulted in the formation of multi-oocyte follicles. Subsequent investigation showed that Rac1 induced nuclear import of STAT3 by physical binding. In turn, nuclear STAT3 directly activated the transcription of essential oocyte-specific genes, including *Jagged1, GDF9, BMP15* and *Nobox*. Further, GDF9 and BMP15 regulated the translation of Notch2 via mTORC1 activation in pregranulosa cells. Overexression or addition of Jagged1, GDF9 and BMP15 not only reversed the effect of Rac1 disruption, but also accelerated primordial follicle formation via Notch2 signaling activation. Collectively, these results indicate that Rac1 plays important roles as a key regulator in follicular assembly.

The primordial follicle pool formed perinatally in mammalian ovaries is the only source of developing follicles and oocytes throughout the entire reproductive life span^1^. In mammals, follicle cohorts are activated with cyclic periodicity from a pool of dormant primordial follicles to produce mature oocytes, resulting in a progressively decreased number of primordial follicles. The general belief is that when the available pool of primordial follicles is depleted, reproduction ceases^2^,^3^. Thus, the size of the primordial follicle pool determines the total fertility of a female.

In mice, primordial germ cells migrate to the urogenital ridge around embryonic day 11 (E11)^4^. By E13.5, germ cells undergo several rounds of mitotic proliferation^5^ and then enter the first meiotic division to become oocytes. These oocytes are directly adjacent to each other in cyst structures that are surrounded by squamous somatic cells (precursor granulosa cells)^6^,^7^,^8^,^9^,^10^,^11^. Near the time of birth, germ cells undergo a wave of apoptosis. Meanwhile, somatic cells move into the nest and intersperse between the remaining oocytes to form primordial follicles. The process of cyst breakdown and primordial follicle formation typically lasts from E17.5 to postnatal day (PND) four^10^,^12^. To date, progesterone and estrogen have been shown to have a negative role on primordial follicle formation^13^,^14^. Numerous studies have demonstrated that this process requires spatiotemporal expression of certain genes. For example, follicle development in GDF9/BMP15 double mutant mice arrests in early folliculogenesis and multi-oocyte follicles (MOFs) subsequently develop^15^. Mutation of Nobox, an oocyte-specific homeobox gene expressed in germ cell cysts and primordial and growing oocytes, delays the breakdown of germline cell cysts^16^. Recent studies have shown that follicular assembly is also regulated by the Notch signaling pathway^14^,^17^. Interestingly, conditional deletion of Jagged1 expressed in germ cells or Notch2 expressed in pregranulosa cells leads to germline cell cysts persistence and formation of MOFs^18^,^19^. However, the molecular mechanisms by which molecule(s) regulate coordinated expression of the essential genes that lead to germline cell cysts breakdown and primordial follicle formation remain unknown. In particular, the relationship between oocytes and pregranulosa cells during this process also needs to be further investigated.

Rac1 belongs to the small (21 kDa) RhoGTPase family that are a ubiquitous protein family^20^ that acts as molecular switches, cycling between an active GTP-bound state and an inactive GDP-bound state. In its active GTP-bound state, Rac1 plays an important role in the regulation of cell shape, adhesion, movement, endocytosis, secretion, and growth^21^,^22^. Several lines of evidence indicate that Rac1 may play a critical role in aspects of tumorigenesis and cancer progression^23^,^24^,^25^,^26^,^27^,^28^ and may function in the terminal differentiation and pigmentation of hair^29^. Recent studies have implicated Rac1 in the regulation of many reproductive events, including meiotic spindle stability and anchoring in oocytes^30^, embryo implantation^31^,^32^, and embryonic epithelial morphogenesis^33^. However, whether Rac1 has a role in early folliculogenesis has not yet been reported. Rac1 affects transcription of many genes via STAT3, a member of the signal transducers and activators of transcription (STAT) family^34^,^35^,^36^. Therefore, we postulated that Rac1 plays a physiological role in primordial follicle formation and modulates transcriptional activation of genes necessary for follicular assembly.

To test this hypothesis, we investigated the physiological role of Rac1 in follicular assembly in mice. We discovered that Rac1 expressed in germ cells regulates primordial follicle formation and controls transcription of essential oocyte-enriched genes, including Jagged1, GDF9, BMP15 and Nobox, by inducing STAT3 nuclear translocation. By regulating Jagged1, GDF9 and BMP15, Rac1 activates the Notch2 signaling pathway in pregranulosa cells. These results indicate that Rac1 is crucial for primordial follicle formation in the mouse ovary under physiological conditions.

## Results

### Rac1 is spatiotemporally expressed in germ cells and directs follicular assembly

**Figure 1:**
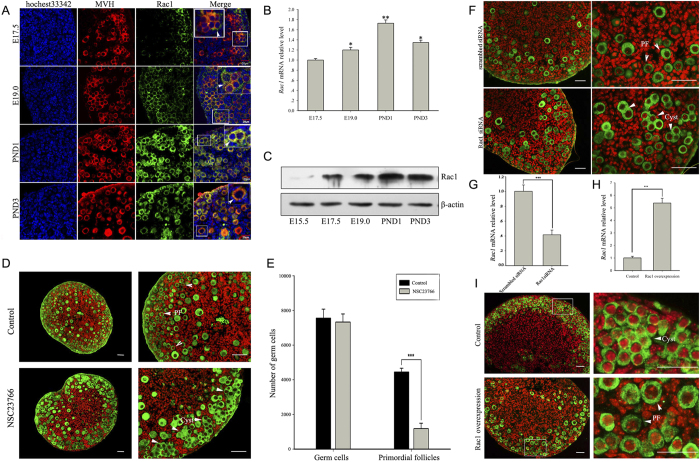
Rac1 is spatiotemporally expressed in germ cells and directs follicular assembly. (**A**) Immunostaining reveals cellular localization of Rac1 in perinatal mouse ovaries. Sections from ovaries on different days labeled for the presence of Rac1 (green), the germ cell marker MVH (red) and the nuclear marker hochest33342 (blue). Scale bar = 20 μm. (**B**) Relative Rac1 expression levels in mouse ovaries on different days measured by RT-qPCR and normalized to β-actin. mRNA levels of E17.5 ovaries were set as 1. Data are expressed as the mean ± s.d., n = 3. P < 0.01 (**), and P < 0.05 (*) versus E17.5. (**C**) Western blot analysis of Rac1 protein levels in perinatal mouse ovaries on different days. β-actin served as a loading control. (**D**) Representative images show retarded primordial follicle formation in NSC23766-treated ovaries compared with controls. Scale bar = 50 μm. (**E**) Whole ovary data from serial sections showed a significant increase in the number of primordial follicles and an unchanged total number of germ cells in NSC23766-treated ovaries compared with controls. P < 0.001 (***) versus the control. (**F**) Representative images show that the Rac1 knockdown slows germline cell cysts breakdown and primordial follicle formation. E15.5 mouse ovaries were treated with Rac1 siRNA or scrambled siRNA for eight days. Scale bar = 50 μm. (**G,H**) Relative Rac1 mRNA levels in control and knockdown or overexpression ovaries measured by RT-qPCR after four days culture. P < 0.001 (***) versus the control. (**I**) Representative images show that Rac1 overexpression accelerates primordial follicle formation. Scale bar = 50 μm.

To investigate the physiological role of Rac1, we first performed immunostaining to determine Rac1 localization in perinatal mouse ovaries. Rac1 was exclusively expressed in germ cells prior to follicle assembly and in the oocytes of primordial follicles (Fig. 1A). Real-time PCR with reverse transcription (RT-qPCR) and Western blot analyses demonstrated that both Rac1 messenger RNA (mRNA) and protein levels were progressively up-regulated during primordial follicle formation (Fig. 1B,C). Expression patterns suggest that Rac1 might have a role in follicular assembly.

To evaluate the physiological function of Rac1, we used the Rac-specific small-molecule inhibitor NSC23766^37^ in an ovary organ culture system. We cultured fetal ovaries (E17.5) with or without NSC23766 for six days (corresponding to postnatal four days) and analyzed ovarian histology. Ovaries treated with 50 μM NSC23766 showed significant inhibition of Rac1 activation after two days in culture (Fig. S1A), and after 6 days culture many germline cell cysts persisted in the cortex compared with controls (Fig. 1D and Fig. S1B). This was supported by additional whole ovarian germ cell and follicle counting data (Fig. 1E). In parallel, we depleted Rac1 by Rac1 siRNA to evaluate specificity. RT-qPCR analyses demonstrated a 55% knockdown (Fig. 1G), and the same phenotype was observed in Rac1siRNA-treated ovaries (Fig. 1F). These results indicate that suppression of primordial follicle formation is caused by a Rac1 deficiency. To further investigate the physiological role of Rac1, fetal mouse (E15.5) ovaries were transfected with a Rac1 overexpression vector and cultured for four days. Rac1 was expressed at levels about five-fold higher than the control (Fig. 1H), and primordial follicle formation was accelerated (Fig. 1I).

To determine whether this effect persisted into puberty, mice were injected with NSC23766 (3 mg/kg·day) or vehicle for three consecutive days from PND0 to PND3. Ovaries from mice treated with NSC23766 exhibited decreased primordial follicle formation at PND3 (Fig. S1C) and an increased incidence of MOFs at PND19 compared with control mice (Fig. S1D) (five vs. zero out of eight mice, respectively). Collectively, these results indicate that Rac1 is spatiotemporally expressed in germ cells and that progressive up-regulated expression facilitates the breakdown of germline cell cysts and assembly of primordial follicles.

### Rac1 regulates the expression of genes necessary for primordial follicle formation

**Figure 2:**
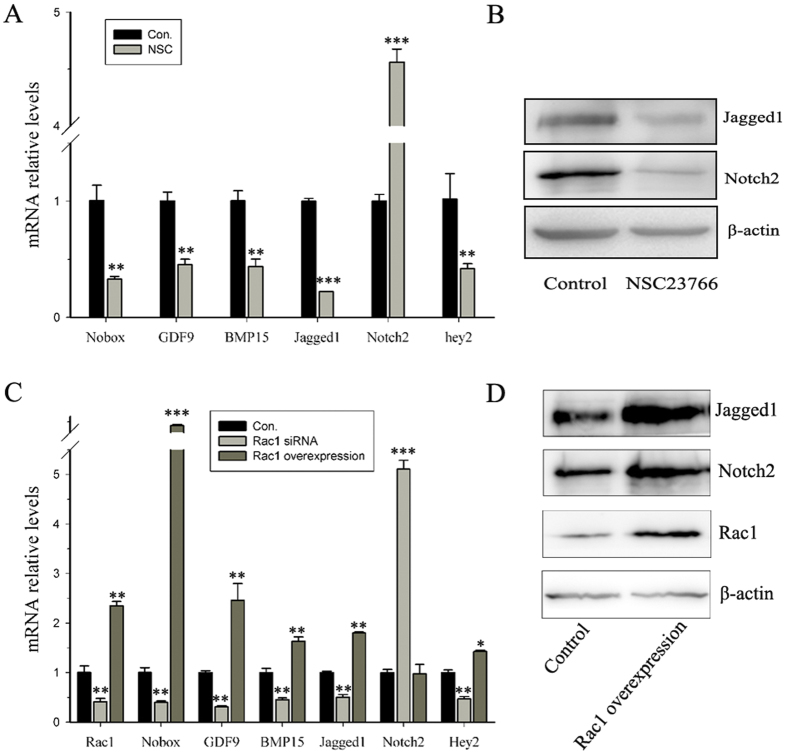
Rac1 directs essential genes expression. (**A**) Relative expression levels of representative genes in germ and pregranulosa cells in control and NSC23766-treated ovaries. mRNA levels were measured by RT-qPCR. mRNA levels in the control ovaries were arbitrarily set as 1. Data are expressed as the mean ± s.d., n = 3. P < 0.001 (***) and P < 0.01 (**) versus the control. (**B**) Western blot analyses show that attenuating Rac1 activity causes a decrease in Jagged1 and Notch2 protein levels. β-actin served as a loading control. (**C**) RT-qPCR showed relative expression levels of essential genes in control, Rac1 knockdown and Rac1 overexpression ovaries cultured for four days. Expression levels were normalized to β-actin. mRNA levels in the control ovaries were set as 1. Data are expressed as the mean ± s.d., n = 3. P < 0.001 (***), P < 0.01 (**), and P < 0.05 (*) versus the control. (**D**) Western blot analyses show that overexpression of Rac1 up-regulates Jagged1 and Notch2 protein expression. β-actin served as a loading control.

To delineate the mechanisms of Rac1 in regulating primordial follicle assembly, we cultured E17.5 ovaries with or without NSC23766 for two days (corresponding to the beginning of primordial follicle assembly), followed by RT-qPCR and Western blot analyses. At this time point, Rac1 activity had already been inhibited, although the same follicular types were present in both control and treated ovaries and histologies were similar. Compared with the control, attenuating Rac1 activity resulted in down-regulation of many oocyte genes essential for primordial follicle formation at the mRNA level, including Jagged1^18^, Nobox^16^, GDF9 and BMP15^15^ (Fig. 2A). We also examined Notch2 expression in pregranulosa cells, which is essential for primordial follicle formation^18^,^19^. Although Notch2 mRNA levels were up-regulated (Fig. 2A), protein levels were significantly decreased (Fig. 2B). Similar results also were observed in Rac1 knockdown ovaries (Fig. 2C).

To explore whether premature Rac1 signaling initiates the expression of genes necessary for primordial follicle formation, we overexpressed Rac1 in E15.5 ovaries. After four days in culture, Rac1 mRNA level was significantly increased compared with control ovaries, and oocyte-enriched genes were up-regulated. Notch2 mRNA level did not change (Fig. 2C), but protein level was significantly up-regulated (Fig. 2D). These results demonstrate that Rac1 facilitates oocyte-enriched gene transcription while regulating Notch2 translation in pregranulosa cells.

To further determine whether Rac1 actions *in vivo* also depends on changes in these essential genes, we administered NSC23766 to PND0 mice. After 16 hours, ovaries were processed for gene expression studies and similar results were observed (Fig. S2). These *in vitro* and *in vivo* studies imply that Rac1 positively regulates expression of genes necessary for primordial follicle formation at different levels, which may contribute to primordial follicle formation.

### STAT3 mediates the function of Rac1 during primordial follicle formation

**Figure 3:**
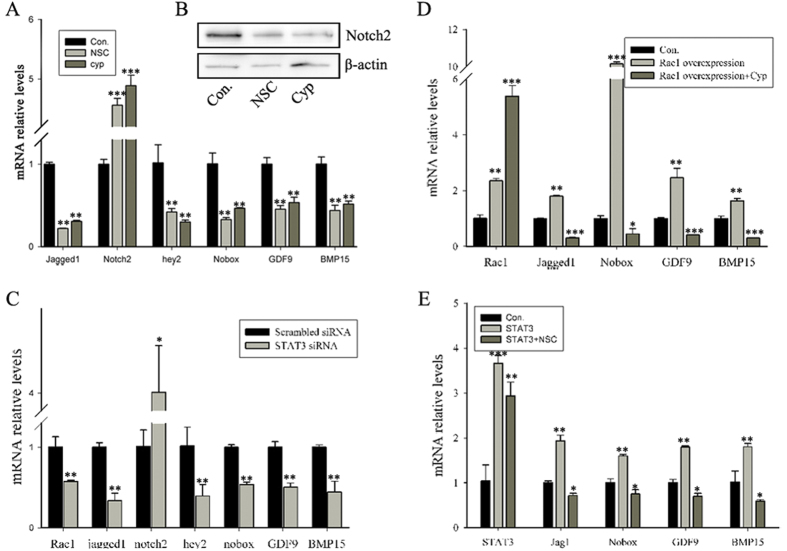
STAT3 mediates the role of Rac1 by affecting essential genes expression. (**A**) RT-qPCR analysis of gene expression in the control and NSC23766-/cryptotanshinone-treated ovaries. Expression levels were normalized to those observed in control ovaries and are represented as the mean ± s.d. (n = 3). (**B**) Western blot analysis of Notch2 protein levels in control and NSC23766-/cryptotanshinone-treated ovaries. β-actin served as a loading control. (**C**) Relative mRNA levels of essential genes in scrambled siRNA- and STAT3 siRNA-treated ovaries measured by RT-qPCR. Relative expression levels were normalized to β-actin. mRNA levels in scrambled siRNA-treated ovaries were set as 1. Values represent the mean ± s.d. from three biological replicates. (**D**) Relative expression levels of oocyte-enriched genes in control, Rac1 overexpression and Rac1 overexpression plus cryptotanshinone-treated ovaries. mRNA levels were measured using RT-qPCR. mRNA levels of control ovaries were set as 1. Data are expressed as the mean ± s.d., n = 3. (**E**) RT-qPCR showed relative expression levels of germ cell-specific genes in control, STAT3 overexpression and STAT3 overexpression plus NSC23766-treated ovaries. Expression levels were normalized to β-actin. mRNA levels of the control ovaries were set as 1. Data are expressed as the mean ± s.d., n = 3. P < 0.001 (***), P < 0.01 (**), and P < 0.05 (*) versus the control.

Many studies have suggested that STAT3 is a potential downstream molecule of Rac1 that delivers Rac1 signaling^36^,^38^. To investigate whether STAT3 mediates the action of Rac1 in follicular assembly, we first examined whether STAT3 has the same spatiotemporal expression and function as Rac1. STAT3 was spatiotemporally expressed in germ cells during follicular assembly (Fig. S3A–C). STAT3 inhibition by cryptotanshinone (a STAT3 selective antagonist) or STAT3 siRNA caused alteration of the same genes (Fig. 3A–C) and led to a persistence of germline cell cysts (Fig. S3D). To confirm whether Rac1 plays a role through STAT3, ovaries (E15.5) overexpressing Rac1 were treated for four days with cryptotanshinone. The compound prevented stimulation of oocyte-enriched genes by Rac1 (Fig. 3D). Similarly, STAT3 overexpression accelerated primordial follicle formation (Fig. S3E) and led to changes in the same genes with Rac1 overexpression, while NSC237766 abandoned the up-regulated genes changes (Fig. 3E). These data suggest that STAT3 mediates the function of Rac1 during primordial follicle formation and that Rac1 affects the role of STAT3 independent of STAT3 expression.

### STAT3 directly binds and activates oocyte-specific genes

**Figure 4:**
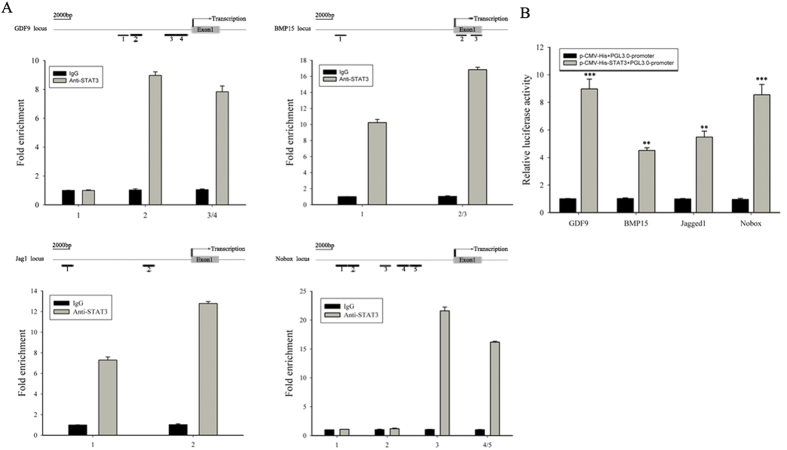
STAT3 directly targets essential oocyte-enriched gene and activates transcription. (**A**) Bottom: ChIP-qPCR analysis demonstrated that STAT3 occupies essential oocyte-enriched gene promoters. Data are presented as fold change compared with IgG enriched DNA fragments. The gene locus and location of various amplicons surrounding transcription start sites are indicated in the diagram at the top of the panel. (**B**) 293FT cells were co-transfected with STAT3 and PGL3-basic-GDF9, BMP15 Jagged1 or Nobox promoters that included predicted STAT3 binding sites. Cell lysates were prepared after transfection and used to measure luciferase activity. Data presented are the mean ± s.d., n = 3. P < 0.001 (***) and P < 0.01 (**) versus the control.

To determine whether STAT3 directly contributes to the activation of oocyte-specific genes, we first investigated whether STAT3 binds to their promoters. Combined with bioinformatic analysis, we performed chromatin immunoprecipitation and quantitative PCR (Chip-qPCR) using ovaries at PND1. Results indicated that STAT3 was enriched in the promoters of essential genes, including Jagged1, GDF9, BMP15 and Nobox (Fig. 4A).

Luciferase assays were also performed to investigate the ability of STAT3 to directly regulate gene promoters. Corresponding promoters (about 2000 bp) containing all STAT3 potential binding sites were cloned into pGL3-basic luciferase reporter vector then were co-transfected into 293FT cells with STAT3 overexpression vector. Results of luciferase reporter assays in 293FT cells showed that STAT3 overexpression significantly enhanced the activity of Jagged1, GDF9, BMP15, and Nobox promoters (Fig. 4B), collectively, these results suggest that STAT3 directly targets and activates transcription of oocyte-specific genes.

### Rac1 physically interacts with STAT3 to facilitate its nuclear trafficking

**Figure 5:**
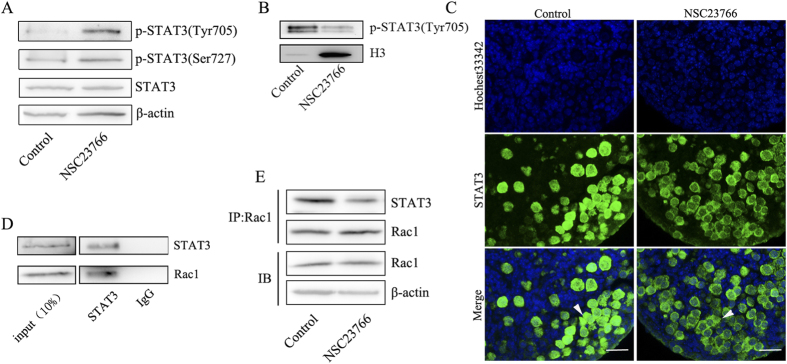
Rac1 induces STAT3 nuclear translocation by direct binding. (**A**) Western blot analysis of STAT3 total protein and phosphorylation levels in control and NSC23766-treated ovaries. β-actin served as a loading control. (**B**) Western blot analyses combined with nuclear protein extraction showed that attenuating Rac1 activity caused a decrease in nuclear p-STAT3 (Tyr705) protein levels. H3 served as a loading control. (**C**) Immunostaining revealed that STAT3 nuclear translocation is dependent on Rac1 activity. E17.5 ovaries were treated with or without NSC23766 for two days. Scale bar = 50 μm. (**D**) STAT3 was co-immunoprecipitated with Rac1 *in vivo*. (**E**) *In vitro* experiment, association between STAT3 and Rac1 was weakened by NSC23766, a Rac1 inhibitor.

To explore how Rac1 regulates STAT3 to stimulate gene expression, we examined whether Rac1 affects STAT3 expression or phosphorylation. E17.5 ovaries were cultured with or without NSC23766 for two days. Western blot showed that attenuating Rac1 activity did not decrease STAT3 expression or phosphorylation at Tyr 705 or Ser727 (Fig. 5A), which induces STAT3 dimerization, nuclear translocation and DNA binding^39^,^40^ and regulates STAT3 transcriptional activation^41^,^42^.

To examine if Rac1 regulates STAT3 nuclear trafficking independent of the phosphorylation at Tyr 705 of STAT3, immunostaining and Western blot combined with nuclear protein extraction were performed. Rac1 inhibition reduced STAT3 nuclear import (Fig. 5B,C), implying that Rac1 regulates STAT3 activity by affecting nuclear import independent of STAT3 phosphorylation levels.

Previous studies have shown that Rac1 directly binds to STAT3 and regulates its nuclear translocation in several human and mouse cell lines^34^,^38^,^43^. To test whether Rac1 regulates STAT3 nuclear transport by direct binding during primordial follicle formation, we used co-immunoprecipitation in ovaries at PND1. Results showed that STAT3 co-immunoprecipitated with Rac1 (Fig. 5D). Moreover, *in vitro* experiments showed that attenuating Rac1 activity significantly reduced the binding (Fig. 5E). These data suggest that active Rac1 regulates STAT3 nuclear trafficking by direct binding.

### GDF9 and BMP15 activate mTORC1 to ensure Notch2 translation in pregranulosa cells

**Figure 6:**
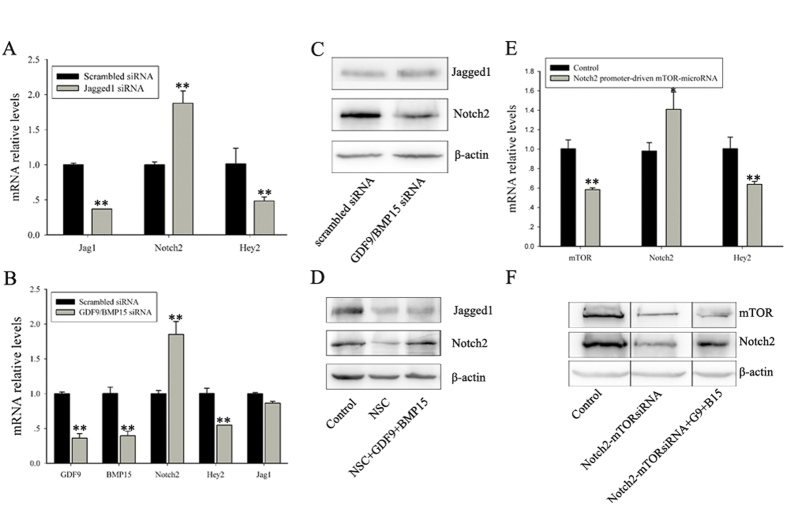
GDF9 and BMP15 mediate the role of Rac1 on Notch2 via mTORC1 in pregranulosa cells. (**A**,**B**) RT-qPCR analyses showed the relative expression levels of Notch2 and hey2 in control, Jagged1 knockdown and GDF9 and BMP15 double knockdown ovaries. Expression levels were normalized to β-actin. mRNA levels of control ovaries were set as 1. Data are expressed as the mean ± s.d., n = 3. P < 0.01 (**) versus the control. (**C**) Western blot analysis of Jagged1 and Notch2 protein levels in control and GDF9 and BMP15 double knockdown ovaries. β-actin served as a loading control. (**D**) Western blot analyses showed that GDF9 and BMP15 purified protein negated the influence of NSC23766 on Notch2 protein levels. E17.5 ovaries were cultured with or without NSC23766 or NSC23776 plus GDF9 and BMP15 for two days. Lysates were subjected to immunoblotting, and β-actin served as a loading control. (**E**) Relative expression levels of Notch2 and hey2 in control and pregranulosa cell-specific knockdown of mTORC1 ovaries. mRNA levels were measured by RT-qPCR. mRNA levels of control ovaries were set as 1. Data are expressed as the mean ± s.d., n = 3. P < 0.01 (**), and P < 0.05 (*) versus the control. (**F**) Western blot analyses show that pregranulosa cell-specific knockdown of mTORC1 caused a decrease in Notch2 protein levels. Addition of purified GDF9 and BMP15 protein recovered the decrease in Notch2 protein. β-actin served as a loading control.

Our results indicate that Rac1 regulates the translation of Notch2 in pregranulosa cells, which prompted us to consider the roles of GDF9/BMP15 and Jagged1 which are essential oocyte genes and may send signals to pregranulosa cells. Previous studies have demonstrated that conditional deletion of Jagged1 or double mutation of GDF9 and BMP15 results in germline cell cysts persistence and MOFs^18^,^44^. To elucidate the underlying mechanisms how oocyte-derived Rac1 regulates the translational alterations of Notch2 in pregranulosa cells,, we transfected E15.5 ovaries with Jagged1siRNA or with a mixed pool of GDF9siRNA and BMP15siRNA. After four days in culture, RT-qPCR and Western blot were performed. Notch2 mRNA levels were significantly increased in Jagged1 siRNA-treated or GDF9/BMP15 siRNA-treated ovaries compared with controls (Fig. 6A,B), although Notch2 protein levels were only significantly decreased in GDF9/BMP15 siRNA-treated ovaries (Fig. 6C). To further detect a relationship between GDF9/BMP15 and Rac1 in regulating Notch2 expression, GDF9 and BMP15 were added in culture and expression of proteins involved in Notch signaling was detected. Compared with the control, 100 ng/mL GDF9 and BMP15 protein largely recovered Notch2 protein levels, but did not have an effect on Jagged1 in NSC23766-treated ovaries (Fig. 6D). The results indicate that GDF9/BMP15 mediated the action of Rac1 to modulate Notch2 at the translational level.

To explore how GDF9 and BMP15 regulate Notch2 translation, we focused our attention on mTORC1, which is regulated by growth factors and cytokines and facilitates protein translation, including Notch2 45,46. To examine if GDF9 and BMP15 affect Notch2 translation via mTORC1, we constructed a pregranulosa cell-specific amiRNA knockdown vector driven by a Notch2 promoter and injected it into E15.5 ovaries. After four days in culture, RT-qPCR and Western blot analyses showed that the mTORC1 knockdown resulted in an increased in Notch2 mRNA levels (Fig. 6E), but a significant decrease in protein level(Fig. 6F). Moreover, additional experiments with GDF9 and BMP15 showed that Notch2 was largely recovered at the protein level (Fig. 6F). These results imply that Rac1 regulates Notch2 translation by GDF9- and BMP15-mediated mTORC1 activation in pregranulosa cells.

### Rac1-mediated up-regulation of Jagged1, GDF9 and BMP15 directs primordial follicle assembly by activating the Notch2 signaling pathway

**Figure 7:**
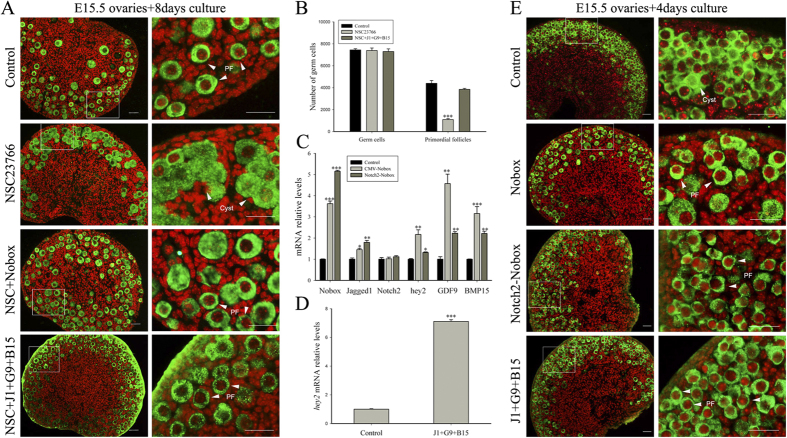
Rac1 directs primordial follicle assembly mainly through Jagged1, GDF9 and BMP15. (**A**) Overexpression of Nobox or addition of purified proteins Jagged1, GDF9 and BMP15 largely rescued the effects of NSC23766 on primordial follicle formation. NSC23766, Nobox expression vector plus NSC23766 or NSC23766 plus Jagged1, GDF9 and BMP15 purified protein-treated fetal mouse ovaries (E15.5) were cultured for eight days. Scale bar = 50 μm. (**B**) Whole ovary data counts by serial sections showed the total number of germ cells and primordial follicles in control and ovaries treated with NSC23766 and NSC23766 plus purified proteins Jagged1, GDF9 and BMP15. P < 0.001 (***) versus the control. (**C**) RT-qPCR shows relative expression levels of GDF9, BMP15 and molecules associated with the Notch2 signaling pathway in control, Nobox widely overexpressed and pregranulosa cell-specific overexpressed ovaries. Expression levels were normalized to β-actin. mRNA levels of the control ovaries were set as 1. Data are expressed as the mean ± s.d., n = 3. P < 0.001 (***), P < 0.01 (**), and P < 0.05 (*) versus the control. (**D**) Relative expression levels of the Notch signaling pathway target gene hey2 in control and purified protein-treated ovaries. mRNA levels were measured using RT-qPCR. mRNA levels of control ovaries were set as 1. Data are expressed as the mean ± s.d., n = 3. P < 0.001 (***) versus the control. (**E**) Representative images show the morphology of the control and CMV and Notch2 promoter driven-Nobox expression vectors and purified Jagged1, GDF9 and BMP15 treated ovaries for four days in culture. Scale bar = 50 μm.

To elucidate if Jagged1, GDF9 and BMP15 regulate primordial follicle formation, we overexpressed Nobox, which facilitates *Jagged1*, *GDF9* and *BMP15* transcription, in E15.5 ovaries for eight days in culture (corresponding to postnatal day 4). Ovarian histology analysis showed that the phenotype caused by NSC23766 was largely reversed. In parallel, fetal mouse ovaries (E15.5) were cultured in medium without treatment as a control and with 50 μM NSC23766 and 50 μM NSC23766 plus 100 ng/ml Jagged1, GDF9 and BMP15 for eight days. After culture, ovaries were fixed and sectioned. Compared with NSC23766-treated ovaries alone, addition of Jagged1, GDF9 and BMP15 reduced the number of germline cell cysts and increased the number of primordial follicles (Fig. 7A). These results imply that Rac1 regulates primordial follicle formation mainly through the secreted proteins Jagged1, GDF9 and BMP15. Results were further confirmed by whole ovary counting data (Fig. 7B).

To explore whether premature expression of Jagged1, GDF9 and BMP15 accelerated primordial follicle formation, CMV or Notch2 promoter driven-Nobox overexpression vectors were transfected into fetal mouse ovaries; or GDF9, BMP15, and Jagged1 were added to the culture system. E15.5 mouse ovaries were cultured for four days, followed by ovarian histology and RT-qPCR analyses. Results showed that both CMV and Notch2 promoter driven-Nobox overexpression vectors up-regulated *Jagged1, GDF9, BMP15* and *hey2* (Fig. 7C) and led to premature germline cell cysts breakdown and primordial follicle formation (Fig. 7E). Addition experiments also resulted in a similar phenotype (Fig. 7E) with an approximately seven-fold increase in *hey2* expression compared with the control (Fig. 7D). Up-regulated Jagged1, GDF9 and BMP15 accelerate primordial follicle assembly and activate Notch2 in pregranulosa cells.

## Discussion

**Figure 8:**
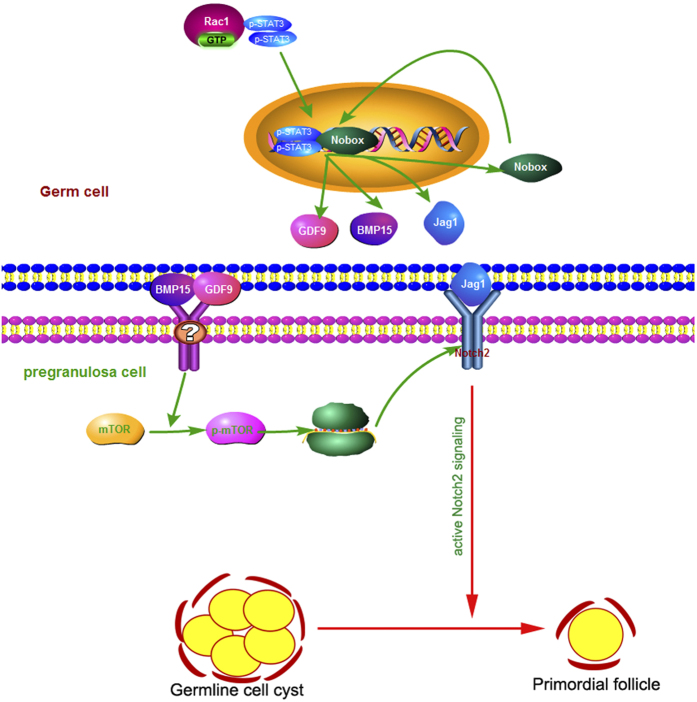
Model for the role of Rac1 in early folliculogenesis. In germ cells, Rac1GTP directly binds to STAT3 to induce its nuclear trafficking. STAT3 is enriched on the promoters of Jagged1, GDF9, BMP15 and Nobox and activates their transcription. Up-regulated transcriptional factor Nobox further enhances the transcription of Jagged1, GDF9 and BMP15. GDF9 and BMP15 bind to the receptor in pregranulosa cells to activate mTORC1, which then facilitates Notch2 translation. Jagged1 binds to Notch2 to activate Notch signaling pathway. Activated Notch2 promotes germline cell cysts breakdown and primordial follicle formation.

In this study, we demonstrated that Rac1 is essential in primordial follicle formation in mice. Our results also suggest a regulatory mechanism to explain how Rac1 facilitates follicular assembly. Based on our data, we propose a model in which Rac1 induces STAT3 nuclear trafficking through direct binding. STAT3 in turn activates *Nobox, Jagged1, GDF9* and *BMP15* in germ cells. Up-regulated Nobox further enhances Jagged1, GDF9 and BMP15 expression. Subsequently, GDF9 and BMP15 modulate Notch2 protein translation by activating mTORC1 in pregranulosa cells. Finally, Jagged1 binds to Notch2 to activate the Notch2 signaling pathway to facilitate primordial follicle formation (Fig. 8).

Rac1 belongs to the Rho family of small GTPases, which are important intracellular signaling proteins^47^. Evidence indicates that Rac1 is involved in many reproductive events, such as meiotic spindle stability and anchoring in oocytes^30^, embryo implantation^31^,^32^,^48^,^49^,^50^, and embryonic epithelial morphogenesis^33^. However, whether Rac1 plays a role in early folliculogenesis has not yet been reported. Using a mouse ovary culture model, we provide evidence that Rac1 is crucial for primordial follicle formation. *In vivo* inhibitor injection experiments showed that attenuating Rac1 activity contributed to germline cell cysts persistence until puberty to form MOFs, which have two or more germ cells trapped within a follicle boundary^51^,^52^. Our results indicate that Rac1 has an important role in early folliculogenesis. However, further investigation using a conditional gene deletion mouse model is needed.

Previous studies have revealed that many genes are essential for germline cell cysts breakdown and follicular assembly, including oocyte-enriched Jagged1, GDF9/BMP15 and Nobox^18^,^19^,^53^,^54^. Pregranulosa cell-specific Notch2 has also been demonstrated to play a crucial role during primordial follicle formation^17^,^19^,^55^. However, the mechanisms by which coordinated expression of essential genes occur are not clear. To explore how Rac1 regulates primordial follicle formation, we investigated the relationship between Rac1 and these genes. Rac1 regulated oocyte-enriched essential genes at the mRNA level, while Notch2 was regulated at the protein level in pregranulosa cells. This evidence suggests that Rac1 may be a crucial regulatory molecule of these essential genes. We conclude that Rac1 regulates germ cell-specific gene transcription via a transcription factor while Notch2 translation is regulated by crosstalk between germ cells and pregranulosa cells.

To elucidate how essential oocyte-enriched genes are regulated at the mRNA level, we focused our attention on STAT3, which can up-regulate transcription of many genes by binding to their promoters^40^. Previous studies have demonstrated that STAT3 is expressed in germ cells during primordial follicle formation^56^. However, whether STAT3 has a role in follicular assembly is not clear. In this study, we provide several lines of evidence supporting a critical function of STAT3 in primordial follicle formation and mediation of Rac1. First, STAT3 is prominently expressed in germ cells and has the same expression pattern as Rac1. STAT3 inhibition slows the breakdown of germline cell cysts and follicular assembly. Second, STAT3 inhibition abandons the function of Rac1 overexpression on essential genes. Third, STAT3, a prominently expressed member of a family of signal transducers and activators of transcription (STATs) in perinatal ovaries (Fig. S2A), directly binds and activates essential genes in germ cells and is dependent on a physical interaction with Rac1 to facilitate nuclear translocation. Interestingly, we observed that the expression of Rac1 was reduced with STAT3 siRNA treatment (Fig. 3C). To this result, we guess STAT3 might have potential binding sites at promoter of Rac1 and regulate its transcription. Although we did not find any existed literatures to support our hypothesis, the authors found the potential binding sites of STAT3 at the promoter of Rac1 through bioinformatic analysis. Further investigation need to be performed for concrete mechanism. Previous studies demonstrated that a *Nobox* knockout decreased *GDF9*, *BMP15* and *Jagged1* transcription and that Nobox directly bound to the *GDF9* promoter to activate its transcription^53^,^57^,^58^. Combining Nobox overexpression and knockdown, we also demonstrated that Nobox regulates *GDF9, BMP15* and *Jagged1* transcription (Fig. 7C and Fig. S4). These results suggest that Nobox, up-regulated by STAT3, may further facilitate transcription of these three genes. Although bioinformatic analysis shows that Jagged1 and BMP15 promoters contain the DNA-binding sequences of NOBOX, whether Nobox directly activates their transcription will require further investigation. Our work uncovered the role of STAT3 in early folliculogenesis and indicates that it mediates the function of Rac1 on primordial follicle assembly.

The concept of crosstalk between germ cells and pregranulosa cells is widely accepted and believed to be important for mediating germline cell cysts breakdown and primordial follicle formation. For example, previous studies in mice with gene mutations demonstrated the importance of germ cell-derived signals in regulating primordial follicle formation, including Jagged1 and GDF9/BMP15 18,54. Jagged1 has been suggested to interact with Notch2 in pregranulosa cells to regulate primordial follicle formation^18^. However, how GDF9 and BMP15 regulate follicular assembly is not clear. Herein, we provide evidence that GDF9 and BMP15 are necessary for Notch2 translation. We demonstrated *in vitro* that GDF9 and BMP15 activate mTORC1 to facilitate Notch2 mRNA translation in pregranulosa cells and mediate the role of Rac1 on Notch2. However, the detailed mechanisms involved will require further investigation.

In the present study, we also provide several lines of evidence to indicate that the oocyte-specific secretory proteins Jagged1, GDF9 and BMP15 mediate the action of Rac1 in regulating primordial follicle formation. First, overexpression of Nobox, which regulates GDF9, BMP15 and Jagged1, eliminated the effect of NSC23766 on primordial follicle assembly, showing that attenuation of Rac1 activity retards primordial follicle formation by affecting these three secretory proteins and also suggests that Rac1 does not affect Nobox nuclear translocation, but rather facilitates Nobox transcription via STAT3. Second, to remove the effects that Nobox may have on other secretory proteins, a protein addition experiment indicated that the three secretory proteins indeed reversed the effect of NSC23766 on follicular assembly. Third, premature expression of GDF9, BMP15 and Jagged1 by Nobox overexpression or addition of purified protein accelerated germline cell cysts breakdown and primordial follicle assembly and activated the Notch2 signaling pathway. A Notch2 promoter driven-Nobox vector, which facilitates GDF9, BMP15 and Jagged1 expression in pregranulosa cells and functions in an autocrine manner, also facilitated follicular assembly and activation of Notch signaling pathway, implying that Rac1 activates the Notch2 signaling pathway by GDF9, BMP15 and Jagged1 independent of gap junctions between germ and pregranulosa cells. Finally, the activation of Notch2 signaling in pregranulosa cells by Jagged1, GDF9 and BMP15 may accelerate primordial follicle formation.

In conclusion, our results demonstrate an important function of Rac1 in regulating primordial follicle formation, which represents a key stage in determining the fertility potential in females. Rac1 likely regulates synergic expression of essential genes and crosstalk between germ cells and pregranulosa cells to facilitate primordial follicle formation. These findings contribute to the overall understanding of the regulatory mechanisms governing the physiological and pathological processes in mammalian ovaries.

## Materials and Methods

### Animal treatment and ovary collection

Adult CD-1 female mice were purchased from the Laboratory Animal Center at the Institute of Genetics in Beijing and bred to male mice of the same strain. Vaginal plug detection was considered E0.5. PND 0 marks the first 12 h after birth. Pregnant mice were housed under controlled lighting (12 h light, 12 h dark) and temperature (21–22 °C) conditions. All experimental procedures were performed in accordance with guidelines approved by the Institutional Animal Care and Use Committee of China Agricultural University. The study was approved by the Institutional Animal Care and Use Committee of China Agricultural University. Ovaries were collected from mice at designated time points. Ovaries were either stored at −80 °C for subsequent RNA isolation and Western blot, immediately fixed in 4% paraformaldehyde for immunohistochemical studies, or prepared for *ex vivo* culture.

### *In vitro* ovary organ culture and chemicals

E15.5 or E17.5 fetal ovaries were collected as described previously^7^ and cultured in 1 mL DMEM/F12 media (GIBCO, Life Technologies, Carlsbad, CA, USA) in a 6-well culture plate (NEST Biotechnology, Beijing, China) at 37 °C in 5% CO2 with saturated humidity. The medium was supplemented with penicillin and streptomycin to prevent bacterial contamination and was changed every other day. Inhibitor-treated ovaries were cultured for two or six days, while siRNA or vector transfected ovaries were cultured for four days or eight days. After culture, ovaries were either stored at −80 °C for subsequent RNA isolation and Western blot or immediately fixed for immunohistochemical studies.

Unless otherwise specified, all chemicals and reagents used in the present study were purchased from R&D Systems, Minneapolis, MN, USA.

### RNA interference (RNAi) and gene overexpression

To ensure that siRNAs and overexpression vectors would be transfected into the inner cells of fetal ovaries, 0.5 μL of the siRNAs or overexpression vectors were first injected into isolated E15.5 fetal ovaries using glass pipettes with a stereomicroscope. After the ovaries were full of liquid, electrotransfection was performed by applying three 5-ms-long quasi-square pulses at a pulse-field strength of up to 30 V/cm. Rac1siRNA and STAT3 siRNA were purchased from Sigma. GDF9 siRNA and BMP15 siRNA pools were chemically synthesized (GenePharma Corporation, China), with sequences listed in Table S1. Control siRNA contained a scrambled siRNA sequence that did not lead to specific degradation of any known mouse mRNA. Ovaries then were cultured for four days to test for transfection efficiency of mRNA and protein levels using real-time PCR and Western blotting, respectively, or for eight days for histological examination and follicle counting.

### Immunohistochemistry

Ovaries were fixed in 4% paraformaldehyde overnight at 4 °C. Before tissue processing, samples were dehydrated through a graded series of ethanols and infiltrated with paraffin. Embedded ovaries were cut into 5-μm thick sections. Sections used for immunohistochemistry were deparaffinized and rehydrated. After washing in phosphate-buffered saline, samples were boiled in a microwave for 16 min in citrate buffer for antigen retrieval. After blocking with 10% donkey serum, the sections were incubated with primary antibody overnight at 4 °C. Antibodies were diluted as follows: Rac1 (Santa Cruz Biotechnology Inc.) 1:50, MVH (Abcam) 1:100, and STAT3 (Cell Signaling Technology) 1:100. Sections were subsequently incubated with Alexa Fluor 488- or 555-conjugated secondary antibodies (1:100, Invitrogen) at 37 °C for 70 min in PBS. The sections were then rinsed with PBS and stained with Hoechst 33342 (B2261, Sigma) for 15 min. Finally, 20 μL Vectashield mounting medium (Applygen) was applied to each slide, and a coverslip was sealed in place. A Nikon 80i was used for imaging immunofluorescent sections, and an Olympus FV100 was used for imaging *in situ* oocyte chromosome analysis. An isotype matched IgG was used as the negative control.

### Histological sections and follicle counts

Ovaries were fixed in cold 4% paraformaldehyde for 24 h, embedded in paraffin, and serially sectioned at 5 μm. The sections were stained with hematoxylin, and the number of oocytes and follicles were counted in every fifth section. To estimate the total number of oocytes and follicles in each ovary, the sum was multiplied by five.

### Real-time RT-PCR analysis

Total RNA was isolated from mouse ovaries with TRIZOL (Invitrogen). One microgram of total RNA was used to synthesize cDNA according to the manufacturer’s instructions (Promega). Real-time-PCR using SYBR green was performed in an ABI sequence detector system according to the manufacturer’s protocol (Applied Biosystems). Expression of individual genes was normalized to the level of β-actin. Three samples at indicated stages were collected and reactions were performed at least twice on each sample. Primers are listed in Table S2.

### Western blot analysis and Rac1 activation assays

Mouse ovaries were lysed with RIPA buffer (9806; Cell Signaling, Danvers, MA, USA) containing 1 mM phenylmethylsulfonyl fluoride (8553S; Cell Signaling). Nuclear protein was extracted using a nuclear extraction kit according to the manufacturer’s instructions (Millipore). Protein concentrations in each group were determined using a bicinchoninic acid assay reagent (Vigorous Biotechnology, Beijing, China) according to the manufacturer’s recommendations. An equal amount of protein was separated by electrophoresis using a 10% PAGE gel. Proteins were transferred onto PVDF membrane and Western blot was performed using the corresponding antibodies against Rac1 (Santa Cruz Biotechnology Inc.); Jagged1, Notch2, STAT3, p-STAT3 (Tyr 705), p-STAT3 (Ser727), mTOR, p-mTOR (Cell Signaling Technology) and β-actin (Sigma-Aldrich). Rac1 activity was determined using pull-down assay kits from Pierce following the instructions^8^. In brief, 50 μg of clarified total lysates were incubated with GST-Pak1-PBD in resins at 4 °C for 1 h in a spin column, centrifuged, and washed three times with lysis buffer to only pull down the active form of Rac1. The samples were boiled and run on a 12% PAGE separating gel and subjected to Western blot analyses using an antibody against Rac1. Rac1 activity was indicated by the amount of Pak1-PBD-bound Rac1 (GTP-Rac1). Total cell lysates were also directly immunoblotted for total Rac1 levels for normalization.

### Co-immunoprecipitation (Co-IP)

Co-immunoprecipitation (Co-IP) experiments were performed on whole ovary protein extracts obtained from 40 ovaries using an immunoprecipitation kit (Life Technologies) according to the manufacturer’s instructions. Antibodies used for IP were anti-Rac1 (Millipore), anti-STAT3 (Cell Signaling), normal rabbit serum, or normal mouse serum (IgG). The precipitated protein complexes were then used for Western blot analysis as described above.

### Chromatin immunoprecipitation (ChIP)

ChIP assays were performed using a MAGNA ChIP kit (Millipore) according to the manufacturer’s protocol. Immunoprecipitations were performed with cross-linked chromatin from PND1 ovaries and either 5 μl of anti-STAT3 antibody (Cell Signaling, 9139) or 1 μL normal IgG. Enriched DNA was quantified by real-time PCR. Primers are listed in Table S3.

### Plasmid construction

Jagged1, GDF9, BMP15 and Nobox promoters were amplified from mouse genomic DNA by the RT-PCR method using specific primers (Table S4). The forward primer contained a Kpn1 restriction enzyme site and the reverse primer contained a HindIII restriction enzyme site. PCR products were purified from agarose gel, digested, and cloned into the Kpn1 and HindIII sites of a pGL3-basic luciferase reporter vector (E1910; Promega). To germ- or pregranulosa cells-specific overexpress or knock down the specified genes, corresponding CDS or synthesized amiRNA cassettes were cloned into MVH (germ cells) or Notch2 (pregranulosa cells) promoter driven expression vectors. Primers are listed in Table S4 and Table S5. All of the constructs were verified by sequencing.

### Transient transfection and luciferase assays

Human embryonic cells (293FT) were cultured in Dulbecco’s modified Eagle’s medium with 10% fetal bovine serum (Invitrogen) supplemented with 100 IU/ml

penicillin and 100 IU/ml streptomycin. 293FT cells were plated at a density of 5 × 104 cells per well in 24-well plates. After 24 hours in culture, cells were transfected with the STAT3 expression vector or the pCMV-C-His control vector, Jagged1, GDF9 or BMP15 or Nobox luciferase reporter vectors, and pTK-Ranilla vector (E2241; Promega) at a ratio of 10:4:1 using the VigoFect transfection reagent (Vigorous Biotechnology). Cells were harvested 24 hours after transfection. Luciferase activity was measured using a dual-luc assay kit (E1960; Promega) on a Modulus™ microplate luminometer (Turner Biosystems, Sunnyvale, CA, USA). All transfection experiments were performed at least three times.

### Statistical analysis

Each experiment was repeated at least three times. Statistical analysis was performed using the SPSS 11.5 program. Comparison of means was performed using the independent samples t test, and data are shown as the mean ± SEM. p < 0.05 was considered significant.

## Additional Information

**How to cite this article**: Zhao, L. *et al*. Rac1 modulates the formation of primordial follicles by facilitating STAT3-directed Jagged1, GDF9 and BMP15 transcription in mice. *Sci. Rep*. **6**, 23972; doi: 10.1038/srep23972 (2016).

## Supplementary Material

Supplementary Information
