# Supplementary material for: Rac1 modulates the formation of primordial follicles by facilitating STAT3-directed Jagged1, GDF9 and BMP15 transcription in mice
Source: Sci Rep. 2016 Apr 6;6:23972. doi: 10.1038/srep23972 (PMC4822123; doi:10.1038/srep23972)
Supplement: Supplementary Information [file srep23972-s1.pdf]

Rac1 modulates the formation of primordial follicles by facilitating STAT3-directed Jagged1, GDF9 and BMP15 transcription in mice

Lihua Zhao[#], Xinhua Du[#], Kun Huang, Tuo Zhang, Zhen Teng, Wanbao Niu, Chao Wang, Guoliang Xia*

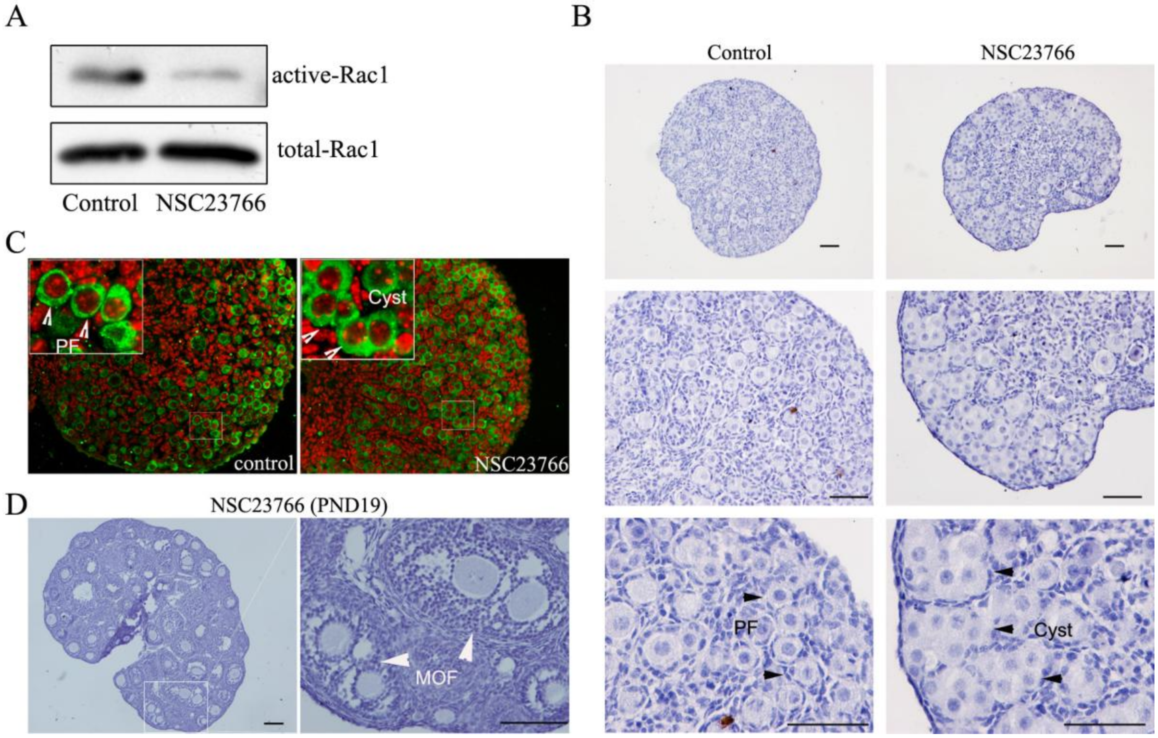

Figure S1 NSC23766 attenuates Rac1 activity and retards germ cell nest breakdown, leading to multi-oocyte follicles

(A) NSC23766 was effective in inhibiting Rac1activation in ovaries. E17.5 ovaries were cultured for 24 h with or without NSC23766. Endogenous Rac1 activation was detected by effector pull-down assays. **(B)** Representative images showed a repressive role of NSC23766 on primordial follicle formation *in vitro*.

E17.5 ovaries were cultured for six days without or with NSC23766. Scale bar=50 μm. **(C)** *In vivo* inhibitor injection experiment showed that attenuation of Rac1 activity hindered primordial follicle formation and **(D)** development into multi-oocyte follicles. Scale bar=100 μm.

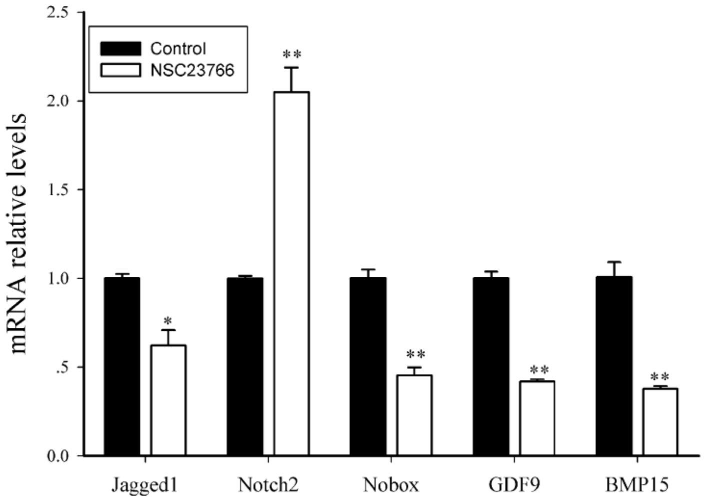

Figure S2 *In vivo* NSC23766 treatment led to changes in gene expression

Neonatal mice were injected with 3 mg/kg d NSC23766 or untreated as controls. After 16 hours, relative mRNA levels were measured by RT-qPCR and normalized to β-actin. mRNA levels observed in the control ovaries were set as 1. Data are expressed as the mean ±s.d., n =3. $P < 0.01$ (**), and $P < 0.05$ (*) versus the control.

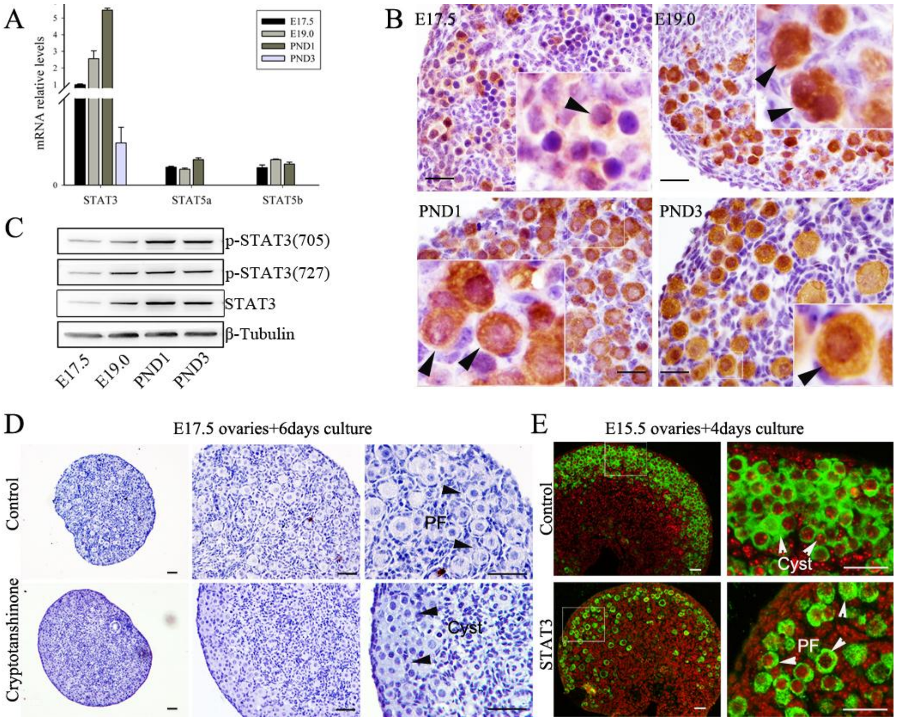

Figure S3 STAT3 is spatiotemporally expressed in germ cells and directs follicular assembly

(A) Relative expression levels of STAT in perinatal mouse ovaries were measured by RT-qPCR and normalized to β-actin. mRNA levels of E17.5 ovaries were set as 1. Data are expressed as the mean ±s.d., n =3. **(B)** Immunohistochemistry results for expression localization of STAT3 in perinatal mouse ovaries. Scale bar=40 μm. **(C)** Western blot analysis of STAT3, p-STAT3 (Try705) and p-STAT3 (Ser727) protein levels in ovaries on different days, with β-actin as a loading control. **(D)** Representative images indicate the phenotypes of the control and STAT3 selective inhibitor-treated ovaries. E17.5 ovaries were cultured for six days, and more germ cell nests were present in treated ovaries. Scale bar=50 μm. **(E)** Immunostaining shows the effects of STAT3 overexpression on primordial follicle formation. E15.5 ovaries were treated with STAT3 overexpression vectors and cultured for four days. Scale bar=50 μm.

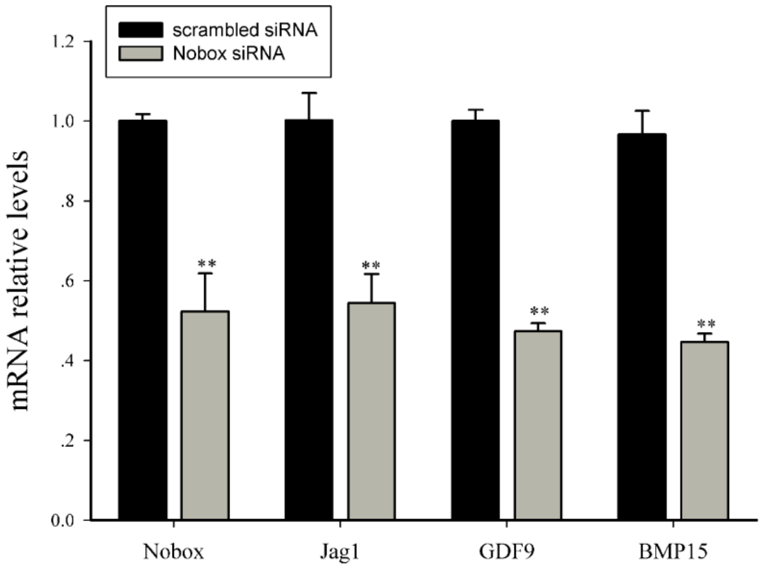

Figure S4 Relative gene expression in control and Nobox knockdown ovaries

E15.5 ovaries were transfected with scrambled siRNA and Nobox siRNA for four days. Relative mRNA levels were measured by RT-qPCR and normalized to β-actin. mRNA levels observed in the control ovaries were set as 1. Data are expressed as the mean ±s.d., n =3. P < 0.01 (**) versus the control.

Supplementary Tables

Table S1 siRNA sequences

**Genes**	**Sequences**
BMP15 sense	CCACUGUGGUUUACCGCCAUCAACU
BMP15 antisense	AGUUGAUGGCGGUAAACCACAGUGG
BMP15 sense	CCGGACCAAGCACUUACCUUCUUCU
BMP15 antisense	AGAAGAAGGUAAGUGCUUGGUCCGG

BMP15 sense	AGAGCCUUGUCAAUGAACUAGUGAA
BMP15 antisense	UUCACUAGUUCAUUGACAAGGCUCU
GDF9 sense	CCGUCCCGUGAAAGAGGAAGCUAUU
GDF9 antisense	AAUAGCUUCCUCUUUCACGGGACGG
GDF9 sense	CAGGUACAACCCUAGGUACUGUAAA
GDF9 antisense	UUUACAGUACCUAGGGUUGUACCUG
GDF9 sense	CACCAUGGUCCAGAAUAUAAUCUAU
GDF9 antisense	AUAGAUUAUAUUCUGGACCAUGGUG

Table S2 Primers for real-time PCR

**Genes**	**Primers**
β-actinF	GTGACGTTGACATCCGTAAAGA
β-actinR	GCCGGACTCATCGTACTCC
Rac1F	ATGCAGGCCATCAAGTGTG
Rac1R	TAGGAGAGGGGACGCAATCT
STAT3F	AGGAGTCTAACAACGGCAGC
STAT3R	ACAGGATTGATGCCCAAGCA
Jagged1F	TGGATTCAAGTGTGTGTGCC
Jagged1R	GGAAGGCAATCACAGTAGTAGC
Notch2F	GCTGTCAATAATGTGGAGGCG

Notch2R	TTGGCCGCTTCATAACTTCC
Hey2F	TGAAGATGCTCCAGGCTACAGG
Hey2R	CCACTTCTGTCAAGCACTCTCG
GDF9F	GATGGGACTGACAGGTCTGG
GDF9R	CAGCGGTCCTGTCACCTG
BMP15F	AAGGGAGAACCGCACGATTG
BMP15R	TGCTTGGTCCGGCATTTAGG
mTORC1-F	AAGCTCTGTTTGTGGCTCTGAA
mTORC1-R	CGCTCTGCTCCTTGATTCTCC

Table S3 Primers for ChIP-qPCR

**Genes**	**Primers**
BMP15-1F	TATGAAGTACCATAAAAGCCAAGG
BMP15-1R	AAGTTATCTTTCCAGCCCCACC
BMP15-2F	ACCTATTAGATTGGGTGCAGGC
BMP15-2R	GGCCAAAGCGAGTCTCCTGACT
GDF9-1F	TTGCTGGGGATTAAATGTAGAC
GDF9-1R	GATTATGTTAGGTAAATTCCGTGA
GDF9-2F	GCAAGATCGGGCCTCAACCTCT
GDF9-2R	TGACTCCAACGGCTCCCTCTGA

GDF9-3F	GCCCTGGGACAGAAGATAGACG
GDF9-3R	CCCTTGAGATCGAAAGAAAATG
Jag1-1F	GAGATGCAGGTAAGAAGTCCAATCA
Jag1-1R	AAAGCATCCCGTTTTCAACATTA
Jag1-2F	GCTCCACGGACATGGATTTGGG
Jag1-2R	GCAGAGGCGACCTGGGCAGACT
Nobox-1F	ACAGCTTCAGCAAAGGGGTCAG
Nobox-1R	CAGCAGCTTATTGGAAGTCACAGATT
Nobox-2F	TTTTACCACCGAGCCATCTCAC
Nobox-2R	AGGTTCTGGACACCAGGCATTT
Nobox-3F	CTCCGGTTGAAGAAGAAGTAAG
Nobox-3R	TATAGACGAGGTTCAAGCGAGT

Table S4 Primers for promoters

**Gene**	**Primer**
BMP15F	CGGGGTACCAGCCAAGGTTCTTGAAAT
BMP15R	CCCAAGCTTGCAATGTAGGGTCGTCAG
GDF9F	CGGGGTACCCCATGTCCTCCTTTCTGACTTTC
GDF9R	CCCAAGCTTTGGTACTGGTCCTTTCCGGCTAC
Jag1F	CGGGGTACCGTTATTGAGCACCCTAACTTGGCGACT

Jag1R	CCCAAGCTTAAGGAACCTGGAAGGACCGTGGA
NoboxF	CGGGGTACCCCTAGTCTATGGCTGGGTATCAGA
NoboxR	CCCAAGCTTTTGTGCCTCAAAGTCCTAACTGA
DDX4F	CCCAAGCTTCCCCAATTTGCTCAGTGGTC
DDX4R	ACGCGTCGACGCTTGGAAGGCAGAGGAGGC
Notch2F	CCCAAGCTTGAAGCACCATGTGGGATGTG
Notch2R	ACGCGTCGACCGCCCGAAGTTTGGCTGAAA

Table S5 Promoters for Gene CDS

**Gene**	**Primers**
Rac1F-BamHI	CGCGGATCCGCCACCATGCAGGCCATCAAGTGTGT
Rac1R-XhoI	CCGCTCGAGCAACAGCAGGCATTTTCTCT
NoboxF-BglⅡ	GGAAGATCTACCGCCATGGAACCTACGGAGAAG
NoboxR-XhoI	CCGCTCGAGTTACTCTTTAGCTCCAGCG
STAT3-BamHI	CGCGGATCCGCCACCATGGCTCAGTGGAACCAGCTG
STAT3R-XhoI	CCGCTCGAGTCACATGGGGGAGGTAGCACA
